# The Role of the Endocannabinoid System in Oncology and the Potential Use of Cannabis Derivatives for Cancer Management in Companion Animals

**DOI:** 10.3390/ani15152185

**Published:** 2025-07-24

**Authors:** Giorgia della Rocca, Alessandra Di Salvo, Erica Salucci, Michela Amadori, Giovanni Re, Cristina Vercelli

**Affiliations:** 1 Department of Veterinary Medicine, University of Perugia, 06126 Perugia, Italy; alessandra.disalvo@unipg.it (A.D.S.); erica.salucci65@gmail.com (E.S.); 2 Research Center on Animal Pain (CeRiDA), University of Perugia, 06126 Perugia, Italy; 3 Department of Veterinary Sciences, University of Turin, 10095 Turin, Italy; michela.amadori@unito.it (M.A.); giovanni.re@unito.it (G.R.); cristina.vercelli@unito.it (C.V.)

**Keywords:** endocannabinoid system, endocannabinoids, phytocannabinoids, oncology, dog, cat, antitumor effects

## Abstract

The last decades of research have shown that the endocannabinoid system may be a promising therapeutic target for the pharmacological treatment of cancer in human medicine and possibly in veterinary medicine as well. Compared with the original cells, the expression of gene encoding for receptors and enzymes belonging to the endocannabinoid system has been found to be altered in several tumor types; it has been hypothesized that this aberrant expression may be related to the course of the neoplasm as well as to the patient’s prognosis. Several studies, conducted both in vitro and in vivo, suggest that both endo- and phytocannabinoids can modulate signaling pathways, controlling cell proliferation and survival. In the complex process of carcinogenesis, cannabinoids seem to intervene at different levels by stimulating cell death, inhibiting the processes of angiogenesis and metastasis, and regulating antitumor immunity. Although the molecular mechanisms by which cannabinoids act are not always clear and defined, their synergistic activity with the most used antineoplastic drugs in clinical oncology is showing promising results, thus providing veterinary medicine with alternative therapeutic targets in disease control. This review aims to summarize current knowledge on the potential role of the endocannabinoid system and exogenous cannabinoids in oncology, with specific reference to the molecular mechanisms by which cannabinoids may exert antitumor activity. Additionally, it explores the potential synergy between cannabinoids and conventional anticancer drugs and considers their application in veterinary oncology.

## Simple Summary

Companion animals are more and more becoming considered family members, and their owners wish to offer them the same level of cure and care expected for a human being. The long life expectancy of dogs and cats is associated with new challenges: veterinary medicine must be prepared to diagnose and treat neoplastic pathology with the same high-standard procedures that are currently used in human medicine. Chemotherapies aim to prolong as long as possible the life of companion animals affected by cancer, but several side effects can be experienced. Thus, an increasing interest in alternative and complementary treatments has arisen in the last years. Among a wide array, cannabinoids seem to be a promising tool to be included in therapeutic protocols since their administration could assist traditional chemotherapeutic agents, promoting a more successful antineoplastic effect, prolonging the prognosis, and contributing to patient well-being thanks to pain relief. According to all the aforementioned factors, the present review aims to summarize how the endocannabinoid system and phytocannabinoids interact in the complex process of carcinogenesis, exploring current therapeutical applications and future perspectives in veterinary oncology.

## 1. Introduction

In recent years, companion animals such as dogs and cats are being increasingly regarded as family members, which has significantly influenced the level of healthcare sought by their owners. Veterinary care has evolved to mirror human medicine, with pet owners now pursuing advanced treatments like surgery, chemotherapy, and physical therapy, which were once reserved only for humans, to ensure their animals’ well-being. In addition, diagnostic strategies have become more sophisticated: veterinarians commonly use blood tests, X-rays, ultrasound, magnetic resonance imaging, and even genetic screening to detect and manage diseases early, just as in human healthcare. This reflects a growing expectation that pets receive the same thorough and compassionate medical attention as people.

The extended life expectancy of dogs and cats due to this paradigm change presents new challenges, as many chronic diseases can affect companion animals at any stage of life, with cancer being one of the most frequently diagnosed conditions. Indeed, in companion animals as in humans, cancer is one of the leading causes of death. Although epidemiological data in veterinary oncology are scarce worldwide, the American Veterinary Medical Association (AVMA) has estimated that approximately one out of four dogs develops a neoplastic disease during its lifetime, with an incidence rate like that reported in humans, and that nearly half of dogs older than ten years die from this disease. The data about the prevalence and incidence of neoplasms in cats are even more limited, but, according to anecdotical experience, neoplasms also represent a significant health concern in feline patients, particularly in older individuals [1].

For historical, epidemiological, and cultural reasons—such as increased life expectancy and close cohabitation with humans in domestic urbanized environments—dogs and cats are the most studied species in veterinary oncology. As stated previously, they also currently benefit from the most advanced diagnostic and therapeutic tools. Even in veterinary medicine, the search for targeted therapies adhering to the concept of “precision medicine” (or rather “precision oncology”) is gaining traction. This approach aims to tailor treatments to individual patients by analyzing the patient–tumor relationship to optimize efficacy and sustainability. Such research, which is beginning to shape modern veterinary oncology, involves genomic studies of neoplasms and the development of alternative and complementary pharmacological therapies that overcome the limitations in available antitumor drugs, limit chemoresistance, and reduce the multiple side effects of commonly used antineoplastic drugs [2]. In this context, the study of endocannabinoids (eCBs) and their phytotherapeutic analogs (i.e., phytocannabinoids, pCBs) has emerged. Over the past two decades, both human and veterinary medicine have focused on the role of the endocannabinoid system (ECS) in the development and progression of various oncological diseases, as well as the possibility of leveraging this system to identify new therapeutic targets.

The ECS can be conceptually defined as a network comprising cannabinoid receptors such as cannabinoid receptor type 1 (CB1R), cannabinoid receptor type 2 (CB2R), G protein-coupled receptor 55 and 119 (GPR55, GPR119), and related receptors such as transient receptor potential vanilloid 1 (TRPV1) and peroxisome proliferator-activated receptor (PPAR). It also includes eCBs such as anandamide (AEA) and 2-Arachidonoylglycerol (2-AG), endocannabinoid-like signaling molecules, proteins involved in their transport, enzymes responsible for endocannabinoid synthesis and catabolism, and the genes encoding these proteins. This system plays a vital role in maintaining homeostatic functions, exhibiting antioxidant, hypotensive, immunosuppressive, anti-inflammatory, and pain-relieving effects. The distribution of cannabinoid receptors in the brain hints at physiological involvement in movement control, perception, sleep, appetite regulation, inhibition of learning and memory, emotional state regulation, and neuroprotection. The ECS affects vasomotor function, fertility, and, last but not least, even tumor cell proliferation [3].

The scientific community unanimously agrees that phytocannabinoid-based therapies provide significant benefits in managing human patients with clinical conditions such as chronic pain, epilepsy, nausea, and appetite loss, among others. From the veterinary side, several studies have been conducted in the last few years in different animal species (mainly in dogs) to confirm the efficacy of cannabis derivatives for the treatment of pain, epilepsy, behavioral disorders, and skin diseases [3,4], underlining the growing interest in the field. However, their potential role as antineoplastic agents remains less clearly defined, especially due to the scarcity of veterinary-specific clinical data compared to human medicine, reinforcing the need for dedicated veterinary research. Nonetheless, an increasing number of in vitro and in vivo studies have demonstrated that cannabinoids—both endogenous and exogenous—can stimulate cancer cell death, inhibit angiogenesis and metastasis processes, and modulate immune mechanisms.

This review aims to summarize the current knowledge on the potential role of the endogenous and exogenous cannabinoids in oncology, with specific reference to (i) ECS modifications in the context of neoplastic diseases, (ii) molecular mechanisms by which cannabinoids may exert antitumor activity, and (iii) the potential synergy between cannabinoids and conventional anticancer drugs. Additionally, (iv) it explores results of in vitro/ex vivo/clinical studies on the antineoplastic effects of cannabinoids published so far in humans, dogs, and cats, and (v) considers their possible application in veterinary oncology.

## 2. Modifications of the Endocannabinoid System in the Context of Neoplastic Diseases

The ECS responds to stressors that alter eCB levels in the body as an adaptive mechanism to restore homeostasis. However, persistent stimuli can impair ECS function, affecting eCB synthesis and the normal expression of its receptors and enzymes. In chronic diseases such as cancer, this dysfunction (probably consequent to genetic mutations, epigenetic changes, and the effects of the tumor microenvironment) appears to contribute to disease progression and the severity of clinical signs [5].

Many scientific studies have explored whether and how the expression of eCBs (CB1R and CB2R) and other ECS-associated receptors varies among different tumor cell lines. Some clinical studies have even investigated whether these components could serve as therapeutic targets [5,6,7]. Unfortunately, the majority of studies present human data, and the relative scarcity of veterinary studies points out the need for more in vitro/in vivo and clinical veterinary research on ECS modifications in the oncology context.

### 2.1. Alterations in Endocannabinoid Ligands

In various human tumor cell lines—including pituitary adenoma, colorectal carcinoma, prostate carcinoma, and endometrial sarcoma—higher levels of AEA and 2-AG have been observed compared to healthy tissues [5]. Regarding nervous system tumors, preclinical and clinical studies have yielded conflicting results: some authors documented higher AEA levels in gliomas and meningiomas than in healthy tissues, while others found a decrease in AEA production in gliomas. Furthermore, 2-AG levels appeared to increase in both tumor types [8].

Regarding studies in animals, Hay et al. [9] compared the levels of various cannabinoids (AEA, 2-AG, oleoylethanolamide—OEA, and palmitoylethanolamide—PEA) among three groups of dogs: one with B-cell multicentric lymphoma, one with T-cell multicentric lymphoma, and a control group (healthy dogs). The study revealed significantly higher levels of OEA, AEA, and PEA in both lymphoma groups compared to the control group, with no correlation found regarding immunophenotype. Conversely, 2-AG levels were higher in healthy dogs. The authors also identified a correlation between patient age and eCB levels: in T-cell lymphoma, these levels appeared to increase with age, whereas, in B-cell lymphoma and the control group, they seemed to decrease. The authors of the study concluded that further studies are needed to explain how eCB concentration varies in different tumors (and immunophenotypes) and, more importantly, to determine their exact role in disease onset and progression [9].

### 2.2. Changes in Cannabinoid Receptor Expression

Similarly, it was reported that the expression of endocannabinoid receptors is modified in cancer cells. In particular, CB1R and CB2R were overexpressed in several human cancers and, depending on the type of neoplasm, this feature was correlated with either poor or better prognosis. Conversely, other studies indicated a downregulation of these receptors in some human cancer types, such as colorectal and hepatocellular neoplasms [10].

A study aimed to evaluate the effects on the ECS of Δ9-tetrahydrocannabinol (THC), a partial agonist of CB1R and CB2R, showed that this pCB increases CB1 gene transcription in both T lymphocyte cell lines and acute lymphoblastic leukemia cell lines. Using a Jurkat T-cell line, it was demonstrated that this induction process occurs through CB2R activation, which mediates IL-4 release and activates the STAT6 transcription factor, necessary for CB1 gene transcription. The gene encoding the CB2R was not differentially expressed following THC stimulation. This study suggests that cannabinoids could induce CB1 gene in cells of the immune system if the above-described mechanism really occurs, thus influencing the immunomodulation of T lymphocytes and their derived tumors [11].

Another ECS-associated receptor, the TRPV1, is physiologically involved in inflammatory responses, pain perception, and calcium metabolism signaling pathways [12,13] Since chronic inflammation is associated with cancer development and aberrant calcium signaling promotes proliferation, metastasis, and tumor cell survival, studies have explored TRPV1’s potential role in tumorigenesis. In vitro, TRPV1 expression has been demonstrated in various neoplasms, including human breast carcinoma, glioma, papillary thyroid carcinoma, and prostate carcinoma [14,15]. Additionally, TRPV1 upregulation has been documented in multiple types of human breast carcinoma compared to healthy tissue, whereas, in murine models, TRPV1 downregulation has been linked to an increased risk of developing colon carcinoma and urothelial carcinoma progression [16].

Furthermore, among the ECS receptors, both CB2R and GPR55 are overexpressed in various tumor cell lines. These receptors can form heteromers with each other, which signal through pathways distinct from those activated by the individual receptors, in order to regulate cell fate. It was speculated that the heteromers could be disease-specific compared to monomeric receptors and a better target for the development of new therapeutic approaches [7,17].

Regarding neoplasm in animals, a study by Rinaldi et al. [18] used immunohistochemistry to demonstrate that CB1R and CB2R are highly expressed in low-grade canine mast cell tumors, at levels like those in healthy mast cells. In contrast, in high-grade mast cell tumors, CB1R and CB2R were scarcely present, indicating a negative correlation between the immunoreactivity of these receptors and tumor aggressiveness [18].

Consistent with these findings, Bulak et al. [19] detected high CB2R immunoreactivity in low-grade cutaneous canine mast cell tumors (grades 1 and 2), reinforcing the hypothesis that less differentiated mast cell tumors exhibit lower CB2R levels. In low-grade cutaneous canine mast cell tumors, the authors also observed a correlation between high CB2R immunoreactivity, the absence or light expression of the p21 protein, and strong immunoreactivity of matrix metalloproteinase-1 (MMP-1). Since this collagenase is supposed to promote the tumor growth during the early stages of tumor progression and cannabinoids have been demonstrated to be able to stimulate the tissue inhibitor of MMP-1, the authors hypothesized that the simultaneous high CB2R and MMP-1 expression suggests a possible use of cannabinoids as an antitumoral target in low-grade cutaneous canine mast cell tumors [19].

### 2.3. Alterations in ECS-Related Enzymes

Finally, the expression of endocannabinoids metabolizing enzymes also resulted in alterations in cancer. AEA and 2-AG are biotransformed by fatty acid amide hydrolase (FAAH) and monoacylglycerol lipase (MAGL), respectively. Studies on gliomas have evidenced that the activity of both enzymes is reduced compared to normal brain tissue. Conversely, other studies reported an increase in these enzymes in other neoplasms such as prostate and pancreas tumors, where the overexpression was correlated to poor and good prognoses, respectively [8].

Results of the cited animal studies are summarized in Table 1.

## 3. Antitumor Mechanisms of Cannabinoids

In the last decades, thanks to the surge in molecular biology and pharmacology techniques, which has enabled more detailed mechanistic insights, a growing amount of preclinical human and animal research aimed at understanding the interplay between the ECS and cancer genesis, with specific regard to the eventual antitumor properties of both eCB and pCB, has been published. Results of these studies are reported in several published reviews [5,7,8,10,16,20,21,22,23,24,25,26,27,28,29,30,31,32,33,34,35,36,37,38,39,40,41,42,43,44,45,46,47,48]. Here, some information specifically concerning the mechanisms by which cannabinoids may exert their antitumor activity are summarized.

Cancer arises as a result of a series of genetic and epigenetic changes: the former involve unrepaired mutations at the DNA level, such as the activation of oncogenes or the inactivation of tumor suppressor genes, while the latter are mostly related to prolonged exposure to agents promoting cell proliferation. The final step is the progression in malignant tumors. Carcinogenesis is a multistep process that develops gradually over long periods and represents the cumulative effect of all these alterations, ultimately leading to the development of neoplasia [49].

In the process of tumor progression, ligands of endocannabinoid receptors, such as AEA, THC, cannabidiol (CBD, another pCB), or synthetic cannabinoid derivatives like WIN 55,212-2 (a non-specific agonist of CB1R and CB2R) and JWH-133 (a selective agonist of CB2R), act at different levels and through multiple signal transduction mechanisms. In recent decades, findings from preclinical studies suggest that cannabinoids can inhibit tumor cell proliferation, neovascularization, invasion, and mechanisms of chemoresistance, in addition to inducing apoptosis and enhancing immune surveillance mechanisms [27].

### 3.1. Induction of Cell Death

Neoplastic cells, by becoming independent to proliferation stimuli control and growth inhibition signals, possess an almost unlimited proliferative potential. Specifically, a loss of sensitivity to pro-apoptotic signals results in an imbalance between cell production and loss, as well as a progressive increase in tumor size and expansion [49].

One of the main antineoplastic effects of cannabinoids appears to be related to the inhibition of proliferation and the induction of tumor cell death through the activation of the autophagy process, leading to subsequent apoptosis. In cannabinoid-induced cell death, autophagy generally precedes apoptosis, but the signaling pathways through which the process is activated vary depending on the specific cannabinoid, the receptor to which it binds, and sometimes the cell type involved. Cannabinoids, specifically CB1R and CB2R agonists (such as AEA and THC), induce the de novo synthesis of ceramide, a sphingolipid directly responsible for increasing the expression of p8 protein, which in turn reduces the intracellular concentration of protein kinase B (PKB), initiating the autophagy process via the mammalian Target Of Rapamycin Complex 1 (mTORC1) inhibition [25,43]. This mechanism has been demonstrated in melanoma, liver carcinoma, and pancreatic carcinoma cell lines, and especially in gliomas. In this last tumor type, it was observed that CB1R agonist binding produces two different (early and tardive) ceramide peaks, and that the apoptosis depends on the second one that is due to the de novo synthesis of ceramide, causing cell death. Conversely, in astrocytes, following the same cannabinoid–receptor interaction, the tardive ceramide peak does not occur, resulting in cell survival [25]. In melanoma, endocannabinoid receptor activation causes the hypophosphorylation of the retinoblastoma tumor suppressor and PKB inhibition, leading to cell cycle arrest at the G1-S transition [50].

Additional intracellular pathways affected by cannabinoids seem to be MAPK/ERK pathways and the modulation of pro-apoptotic/anti-apoptotic proteins (Bcl-2 family). In human colorectal adenoma and carcinoma cells, THC treatment resulted in the CB1-mediated inhibition of both RAS-MAPK/ERK and PI3K-AKT survival signaling cascades, two key cell survival pathways frequently deregulated in colorectal tumors. The inhibition of ERK and AKT activity by THC was accompanied by the activation of the proapoptotic BCL-2 family member BAD. The reduction of BAD protein expression by RNA interference rescued colorectal cancer cells from THC-induced apoptosis. These data suggest an important role for CB1 receptors and BAD in the regulation of apoptosis in colorectal cancer cells [51].

Another extensively discussed and currently recognized mechanism of cell proliferation inhibition involves the ability of non-psychoactive cannabis components (primarily CBD) to induce the production of reactive oxygen species (ROS) and drive cells toward oxidative damage-induced death. This effect does not occur via interaction with CB1R and CB2R but rather through other receptor types, such as TRPV1 (where it acts as an agonist) and GPR55 (where it acts as an antagonist) [25,43].

Additionally, it has been demonstrated that CBD can indirectly promote cell death via an entourage effect: by inhibiting the fatty acid amide hydrolase (FAAH) enzyme, it increases the available concentration of AEA, thereby enhancing the autophagy process [25,43].

Cannabinoids, particularly AEA, can also regulate specific cell cycle checkpoints and prevent tumor progression: AEA can induce cell cycle arrest at the G1-S transition by upregulating cyclin-dependent kinase inhibitors p21 and p27 and reducing the percentage of cells in the G2-M phase [27,35].

Despite the numerous studies supporting the antiproliferative effects of cannabinoids, some in vitro and murine studies have reported the involvement of the ECS in tumor progression. For instance, AEA at a concentration of 1 μM has been shown to stimulate gastric adenocarcinoma proliferation. Similarly, at lower concentrations, its synthetic analogous AM-356 has increased mitogenic activity in prostate carcinoma cells. Likewise, in the same tumor cell lines, THC and JWH-133 have exhibited proliferative effects, which, in the case of THC, have also been observed in lung, breast, and nervous system cancer lines. This pro-cancerous activity is believed to be linked to the role of CB2R in immune response modulation: through CB2R activation, THC appears to increase IL-4 and IL-10 production, which in turn inhibits the Th-1 response and stimulates the Th-2 response [8] (discussed later).

Unfortunately, to the authors’ knowledge, no published research has directly compared different doses of cannabinoids and different contexts with regard to the antitumorigenic or pro-tumorigenic potential of cannabinoids, making it difficult to speculate on dose- and context-dependent biphasic effects (i.e., how low and high doses can produce opposing effects). This deficiency highlights the need for further studies, the results of which could provide insights into careful therapeutic dosing and patient-specific considerations.

It should also be emphasized that cannabinoid effects differ by involved receptor subtype (CB1R, CB2R), receptor heteromers, and non-cannabinoid receptors (TRPV1, GPR55), having different influence on antitumor effects.

### 3.2. Inhibition of Angiogenesis, Invasion, and Metastasis

The invasiveness of solid tumors is supported by the process of angiogenesis, a complex mechanism of neovascularization stimulated and maintained by transformed cells, allowing them to receive oxygen and trophic factors through the creation of a newly formed vascular network that surrounds and thus nourishes the tumor. Angiogenesis is induced by the release of angiogenic factors by the neoplasm, such as Vascular Endothelial Growth Factor (VEGF) and Fibroblast Growth Factor (FGF). Many neoplasms are capable not only of autonomously producing angiogenic molecules but also of inhibiting the synthesis of those with anti-angiogenic activity, such as thrombospondin. Furthermore, neovascularization is fundamental for metastatic dissemination and, therefore, for the metastasis process [49].

Similarly to angiogenesis, the process of lymphangiogenesis allows for the creation of new lymphatic vessels, which also originate through sprouting and allow, especially in lymphatic tumors, for metastatic spread to regional lymph nodes. In this case, factors stimulating vascularization, such as VEGF, also play a crucial role [49].

The anti-angiogenic and anti-metastatic activity of cannabinoids primarily manifests through the reduction in the transcription (downregulation) of genes that encode metalloproteinases, the VEGF factor, and its receptors VEGFR1 and VEGFR2, a process demonstrated in gliomas and skin and thyroid carcinoma. Moreover, cannabinoids have been shown to inhibit the migration of endothelial cells and induce apoptosis, thereby compromising the tumor angiogenesis process. In cell lines of breast, lung, and cervical carcinoma and glioma, they have been shown to inhibit tumor invasiveness and, in animal models, to reduce the process of metastasis [25,32,43].

The study by Blázquez et al. [52] was the first to demonstrate in vivo the anti-angiogenic activity of cannabinoids. Immunohistochemical analyses and vascular permeability assays showed that the administration of JWH-133 to mice with induced gliomas can lead to a change in the characteristic traits of newly formed capillaries, which become differentiated and impermeable. This is associated with the reduced expression of VEGF and angiopoietin-1, another pro-angiogenic factor. Furthermore, the activation of cannabinoid receptors in vascular endothelial cells inhibited cell migration and the expression of MMP-2, a proteolytic enzyme that allows for the disaggregation and remodeling of tissues during angiogenesis and metastasis [25,52].

Similar to the molecular mechanisms mentioned in the previous paragraph, the anti-angiogenic effect of cannabinoid receptor agonists also occurs through the induction of intracellular ceramide accumulation, which activates metabolic pathways linked to the increased expression of the p8 protein. The inhibitory action on tumor angiogenesis has also been observed in studies with CBD, which appears to downregulate the Id-1 (inhibitor of differentiation) protein associated with the aggressiveness of breast cancer and to induce ICAM-1 (intercellular adhesion molecule 1) that are connected to the activity of TIMP-1 (tissue inhibitor of matrix metalloproteinases-1), responsible for the reduction of cell invasiveness in lung cancer [25,32,43].

Despite the anti-angiogenic properties that have been demonstrated and supported by numerous preclinical and clinical studies, over the past twenty years, it has also been hypothesized that some endocannabinoid compounds could, conversely, stimulate angiogenesis through the activation of the GPR55 and CB1R. Among these, AEA has promoted angiogenesis stimulated by FGF-2 through the activation of the CB1R, which has been found to be overexpressed during the angiogenesis process. Some studies conducted on CB1R knockdown mice have reported an inactivation of proliferation, migration, and capillary formation induced by pro-angiogenic factors (particularly FGF-2), but, at the same time, there are also studies on murine models of colon cancer where the loss or inhibition of CB1R has induced tumor growth. Therefore, there is reason to believe that the expression and specific function of these receptors may vary based on the type of neoplasia considered [8].

### 3.3. Regulation of Antitumor Immunity

Carcinogenesis is a pathological process that arises from the organism’s inability to develop an effective immune response against mutated cell antigens. This process typically develops through the mechanism of immune surveillance, which allows components of the immune system to recognize non-self-antigens potentially presented by foreign, transformed, or infected cells. Innate immunity acts as the first line of defense against transformed cells, determining an immediate, nonspecific, and short-lived inflammatory response. Subsequently, acquired immunity develops. Acquired immunity consists of a humoral component that utilizes antibodies (produced by plasma cells derived from activated B lymphocytes) and a cell-mediated component, whose effectors are primarily CD8+ and CD4+ T lymphocytes that differentiate into cytotoxic T-cells and T-helper (Th) cells, respectively. Although tumors can induce an immune response, the effectiveness of this response depends on both the reactivity of the host and the characteristics of the tumor antigens [49].

Since the immune system shapes the tumor microenvironment and has a significant impact on the carcinogenesis process, current therapeutic approaches, including studies on the immunological activity of cannabinoid compounds, increasingly focus on targeting immune system cells [31].

One of the mechanisms for which the endocannabinoid system is currently under extensive research is its potential role in tumor immuno-surveillance, particularly regarding the CB2R, which is primarily expressed on the surface of immune system cells. Since most cells in the tumor microenvironment possess cannabinoid receptors, it remains unclear whether their activity may promote tumor development or exert antitumor effects, especially concerning their immunomodulatory effects [35].

The immune cells with the highest levels of CB2R expression are B lymphocytes, followed by natural killer cells, polymorphonuclear granulocytes, and T lymphocytes, while CB1R is expressed in smaller quantities on these cells. The level of CB2R expression has been shown to correlate with cellular activation status, as well as the presence of immunomodulatory molecules [31,43]. Also, TRPV1 is expressed on macrophages, dendritic cells, T lymphocytes, and natural killer cells, even if research on its role in immunity has given contradictory results [16].

The most widely examined immunomodulatory effects of cannabinoids in the context of cancer concern changes in T-cell activity. Typically, Th-1 lymphocytes synthesize and secrete IL-2 and IFN-γ, stimulating cytotoxic T lymphocytes and macrophages to produce a cell-mediated immune response. In contrast, Th-2 lymphocytes synthesize and secrete IL-4, IL-5, IL-6, and IL-13 to stimulate the transformation of B lymphocytes into plasma cells, thus producing an antibody response. At the same time, they have been shown to inhibit macrophage functions. The regulatory mechanisms of Th-1 and Th-2 lymphocyte responses are believed to be linked to the production of two types of interleukins: while IL-12 produced by macrophages stimulates a Th-1 response, the production of IL-4 promotes the Th-2 response, inhibiting the Th-1 response. It is hypothesized that a Th-1 response is crucial for an effective immune defense against many tumors, as the activation of this type of lymphocyte is necessary for that of cytotoxic T lymphocytes and macrophages. Because cytokines such as IL-2 and IFN γ can promote the response of Th1 lymphocytes while IL-10 suppresses it, the cytokine profile expressed in the tumor microenvironment is significantly important in determining the antitumor response [43].

The activation of CB2R by agonistic molecules, such as THC, can lead to changes in the types of cytokines released by cells. The action of THC has been linked to a shift in Th cell subtypes from a Th-1 profile to a Th-2 profile and a decrease in the production of Th1 cytokines (IL-2, IL-12, and IFN-γ), as well as a decrease in the expression of their receptors. At the same time, THC increases the production of Th2-promoting cytokines (e.g., IL-10 and transforming growth factor-β) that are considered to interfere with immune responses against tumors. Thus, studies seem to indicate that THC, by promoting the Th-2 response, has an immunosuppressive effect [43].

Similarly, CBD, a non-psychoactive cannabinoid with low affinity for the CB2R, has also demonstrated an immunomodulatory activity. For instance, CBD has been shown to suppress T-cell function and inhibit IL-2 production, indicating a potential immunosuppressive activity that requires further investigation [43].

However, there are also numerous studies demonstrating the antitumor activity of these compounds. For example, Haustein et al. [53] showed that a significant part of THC’s antitumor action relates to the modulation of ICAM-1 expression on lung cancer cells. Both THC and CBD, as well as methanandamide (an AEA analogue), have been shown to promote the expression of ICAM-1 on lung cancer cell lines, resulting in an increased susceptibility of tumor cells to lysis mediated by lymphokine-activated natural killer cells [53].

Among the antitumor mechanisms mentioned in this paragraph, the immunoregulatory activity of cannabinoids is undoubtedly the one that requires further investigation and exploration, in the attempt to better explain the dual role of cannabinoids: while they may suppress immune responses that facilitate tumor control (via Th1 inhibition), they can also enhance tumor cell susceptibility to immune killing via ICAM-1 upregulation. Results could help to understand whether and how these opposing effects might be balanced or targeted therapeutically.

## 4. Synergy Between Cannabinoids and Antitumor Drugs

Pharmacologic synergy refers to the interaction between two or more drugs that results in a combined effect greater than the sum of their individual effects. This synergy can lead to enhanced therapeutic efficacy, allowing for lower doses of each drug, which may in turn reduce side effects and improve the therapeutic index (i.e., the ratio between a drug’s effective dose and its toxic dose). Pharmacologic synergy is often exploited in treatments for infections, cancer, pain, and other complex conditions.

As already stated in the introduction, research into targeted therapies adhering to the concept of “precision medicine” is also spreading in the field of veterinary oncology. This modern approach includes, among others, the search for complementary pharmacological therapies that limit chemoresistance phenomena and reduce the side effects of the most used antineoplastic drugs [2].

Chemotherapy regimens that involve the combination of multiple drugs with different antiproliferative activities enhance the organism’s response to cancer treatment. In this context, research introducing cannabinoids into the most common antitumor pharmacological protocols is significantly increasing and showing promising results. Cannabinoids can be combined with the most employed antineoplastic drugs in clinical oncology to increase the sensitivity of cancer cells, thus making these drugs more powerful and allowing one to lower their dosage, consequently reducing potential toxic effects on the organism. Research on the possible synergistic activity between cannabinoids and cytostatic drugs is showing excellent results. For instance, THC and CBD have been found to enhance the cytotoxic impact of various chemotherapeutics such as cytarabine, doxorubicin, mitoxantrone, carmustine, temozolomide, bortezomib, carfilzomib, and cisplatin in preclinical studies [28].

Currently, gliomas are the neoplasms in which the role of the ECS has been most investigated at the preclinical level, particularly within polychemotherapy regimens. For example, in human glioblastoma cells, cannabinoids have been shown to enhance the chemotherapeutic activity of drugs like doxorubicin, carmustine, and temozolomide, reducing the resistance of transformed cells to cell death by apoptosis, neoangiogenesis, and tissue invasion [54]. There are also preclinical studies where the combined administration of THC and CBD with temozolomide resulted in a significant reduction in tumor growth, both in mice with temozolomide-sensitive glioblastoma and those with temozolomide-resistant glioblastoma. Specifically, in a murine xenograft model of glioblastoma, the therapeutic association between THC and temozolomide demonstrated a strong increase in autophagy compared to treatment with temozolomide alone, even in glioblastomas that had previously been resistant to this drug, without showing any side effects [55]. In human glioma cell lines, CBD has induced Ca^2+^ influx through the activation of the TRPV2 receptor channel, increasing the absorption of chemotherapeutic drugs such as doxorubicin, temozolomide, and carmustine [40].

Regarding other cancer types, there are data from studies conducted on breast adenocarcinoma cells using different synergy quantification models that show significant synergistic interactions between CBD and chemotherapeutic drugs such as docetaxel, doxorubicin), paclitaxel, and vinorelbine [56].

THC and CBD have also been shown to enhance the cytostatic effect of vinblastine in human and murine leukemia cell lines resistant to vinblastine through the downregulation of P-glycoprotein, while a similar effect was observed with the simultaneous administration of THC and vincristine in leukemia lines [27].

An experimental study by Punzo et al. [57] investigated the pharmacological association between bortezomib and two selective agonists—one for the CB2R (JWH-133) and one for the TRPV1 receptor (RTX)—in human osteosarcoma (OS) cell lines. The aim was to determine whether a synergistic interaction between proteasome inhibitors and the ECS could be exploited to reduce the side effects of bortezomib in treating human OS (which primarily affects children and adolescents). The results showed that combining bortezomib with RTX increased apoptotic signaling, including caspase-3 expression, a key effector in the apoptotic cascade. Additionally, the combination with JWH-133 positively influenced not only the signaling pathways leading to transformed cell death but also bone metabolism itself [57]. Moreover, in 2023, Li et al. [58] evaluated the synergistic anticancer effect of CBD and doxorubicin in two OS cell lines and found that the CBD/doxorubicin combination treatment synergistically inhibited growth, migration, and invasion and induced apoptosis, blocking G2 stagnation in OS cells, thus suggesting that CBD and doxorubicin have a synergistic anticancer effect on OS cells and their combined application may be a promising treatment strategy for OS [58].

Two studies have evaluated the effects of a combination therapy with CBD and chemotherapeutic agents in different canine tumor cell lines. A study by Henry et al. [59] investigated the possible pro-apoptotic and antiproliferative response of CBD in canine osteosarcoma, lymphoma, and mammary adenocarcinoma cell lines, both alone and in combination with two different drugs, doxorubicin and vincristine. The results of this research showed that the combination of vincristine and CBD consistently enhances the decrease in cell viability compared to a single treatment, indicating a significant synergistic response. In the case of the combined treatment of doxorubicin and CBD, it is interesting to note that different results emerged depending on the specific concentrations of each compound used: according to this study’s results, it seems that higher doses of doxorubicin and CBD yield a synergistic response of the two molecules, while lower doses result in an antagonistic action [59]. Inkol et al. [60] tested the in vitro effects of CBD on the proliferation and apoptosis of canine urothelial carcinoma cells, both as a single agent and in combination with various chemotherapeutic agents (carboplatin, vinblastine, mitoxantrone, and piroxicam). The combination of CBD, mitoxantrone, and vinblastine produced a significant reduction in cell viability and an increase in the apoptosis of neoplastic canine urothelial cells compared to treatment with individual antitumor agents. In contrast, the combination of CBD and carboplatin did not lead to any reduction in cell viability or increase in apoptosis compared to treatment with the single chemotherapeutic agent. Finally, the combination of CBD and piroxicam did not significantly influence cell viability, although some cell lines showed a reduction in cell viability when mitoxantrone was combined with piroxicam [60]. Given these promising results, it is plausible that these interactions could be exploited to treat canine tumors.

From what has been reported, it seems that cannabinoids enhance the efficacy of chemotherapy (e.g., by sensitizing tumor cells, overcoming drug resistance, modulating apoptosis pathways, or affecting the tumor microenvironment). However, the main limitation regarding this subject is that the data are only preclinical, as no clinical trials are available so far (neither in human nor in veterinary medicine). Further studies, specifically concerning dogs and cats, are thus warranted, involving different tumor types and heterogeneity as well as different cannabinoid formulations and dosing. Due to the inhibition of CYP450 enzymes by cannabinoids, studies should also exploit the possible pharmacokinetic interactions between cannabinoids and chemotherapeutics.

## 5. Preclinical and Clinical Studies on the Antineoplastic Effects of Cannabinoids

### 5.1. Preclinical Studies

The currently available human and animal data on the antineoplastic effects of cannabinoids mostly refer to preclinical studies conducted on cell cultures [50,61,62,63,64,65,66,67,68,69,70,71]. Studies have involved different cannabinoids (CBD, THC, cannabichromene, and synthetic cannabinoids) applied on different cell lines (e.g., head and neck squamous cell carcinoma, urothelial cell carcinoma, melanoma, colorectal adenocarcinoma, glioma, prostate cancer, non-Hodgkin’s lymphoma, osteosarcoma, pancreatic ductal adenocarcinoma, among others), demonstrating antitumorigenic activity in most, but not all, instances.

Specifically regarding studies conducted on animals, only three studies conducted in canine species have been published so far. In a study by Gross et al. [69], canine glioma cell lines were treated with CBD extracts and purified CBD to evaluate potential cytotoxicity: purified CBD reduced proliferation and induced cell death via caspase activation [69]. Omer et al. [70] evaluated the effects of CBD, THC, and WIN 55-212-22 on the viability of canine non-Hodgkin’s lymphoma cell lines using malignant canine B-cell lymphoma (1771 and CLB-L1) and T-cell lymphoma (CL-1) cell lines and found that, based on the IC50 values, CBD was the most potent pCB in reducing lymphoma cell viability in 1771 and CL-1 cell lines [70]. The in vitro antitumor effect on canine prostate carcinoma cell lines of cannabis extract oils rich in CBD and THC was studied by Calheiros et al. [71]: in this study, CBD- or THC-rich extracts inhibited the proliferation of two canine prostatic carcinoma cell lines, PC1 and PC2, showing an IC50 of 3.43 and 3.57 μM for CBD-rich extracts, and 4.90 and 4.48 μM for THC-rich extracts [71].

Given the complexity and variability of the functioning of various tumor types, especially regarding the characteristics of the tumor microenvironment, in vitro results are frequently not directly translatable to in vivo conditions. For this reason, research in recent years has focused on the use of pCB in carcinogenesis in animal models, such as those with implanted or grafted tumors and genetically modified animals, as they can generate relatively predictable tumor growth [43,50,68,72,73]. Trials have involved various types of tumors, including those of the nervous system, digestive system, breast, prostate, lungs, thyroid, and skin. Results have shown, depending on the model and the substance tested (eCB, pCB, synthetic cannabinoids), a reduction in tumor mass, slowdown in tumor growth, decreased angiogenesis, reduced metastasis, and increased survival of treated subjects. In some cases, however, effects promoting tumor growth have been observed, probably due to the potential immunosuppressive role that cannabinoids have shown in certain neoplasms.

### 5.2. Clinical Studies

In human medicine, there are publications from clinical trials devoted to determining the effects of cannabinoid formulations, to attempt to understand whether these molecules are effective in treating cancer and to what extent, even in complex organisms [6,43,72,73,74]. Research conducted so far has involved a limited number of patients with various types of tumors (such as recurrent glioblastoma, astrocytoma, lymphoblastic leukemia) refractory to conventional treatments, and various cannabinoids (such as THC and nabiximols) have been administered through different routes (i.e., oral, inhalation, intratumorally, intracranial). Some patients have shown a reduction in tumor growth due to decreased cell proliferation and increased apoptosis, or even tumor regression, that was in some cases dose-dependent. No significant side effects were reported in these studies. However, there are still no definitive data proving the antitumor efficacy of cannabinoids.

The route of administration is the most important factor considered in these trials, as the bioavailability of cannabinoids seems to depend directly on the method of administration: ingestion leads to only 6–20% of all active compounds reaching the bloodstream, while inhalation allows for 10–60% to become bioavailable. The onset time and duration of effects also vary depending on the method of administration: inhalation produces effects within a few minutes but lasting only a couple of hours, while oral administration has a slower onset, between 60 and 90 min, with effects lasting for 6–8 h. Consequently, a direct comparison of the efficacy of a specific formulation is only possible when the route of administration is overlapping [33].

In 2024, the American Society of Clinical Oncology (ASCO) published the ASCO guidelines on the use of cannabis and cannabinoids in human adult patients with cancer. Outcomes of interest included antineoplastic effects, cancer treatment toxicity, symptoms, and quality of life. The evidence base consisted of 13 systematic reviews and 5 additional primary studies (4 RCTs and 1 cohort study). The certainty of evidence for most outcomes was low or very low [75].

One of the main limitations of clinical studies is the very small number of patients involved. So far, no results have been replicated in more patient groups, and the methods and objectives of various studies are often different, making direct comparisons difficult. Despite these limitations, the results obtained nonetheless contribute to providing insights into the possible antitumor role of cannabinoids, a field of study that requires further exploration and the involvement of a larger number of patients. If combined with data from studies on animal models, where the results appear more consistent, such conclusions suggest a possible therapeutic role of cannabinoids in tumor treatment [76].

In veterinary medicine, there is a very limited number of published clinical studies, and the eventual efficacy of cannabinoids in the treatment of oncological patients is mostly anecdotal.

Regarding published data, Buranakarn [77] described the case of a stray cat with a sarcoma located at the eye bulb: as a first approach, a surgical resection was attempted, which failed because, after a few weeks, the mass reappeared, reaching a size of 4 cm in diameter. At this point, the attending veterinarian opted for a palliative approach, prescribing exclusively an oral cannabis oil in the attempt to reduce the patient’s pain. In the following two weeks, the mass decreased in size (a reduction from 5 to 1.5 cm was reported), suggesting that cannabis extracts may have played a role in the reduction of the mass [77]. Hazzah [78] published a case report on the use of medical cannabis as palliative care in a feline with advanced cancer: a 10-year-old 2.2 kg male neutered domestic shorthair feline was presented with a history of dyspnea, coughing, and lethargy; radiographs revealed a large mass encompassing the majority of the cranial thorax and mid-thorax. The implementation of a complex-spectrum cannabis product was well-tolerated and provided a resolution of cancer-related clinical signs for 6 months [78].

Thus, despite promising preclinical results, clinical validation remains limited, and there is an urgent need for well-designed clinical trials to assess the efficacy, safety, and dosage of cannabinoids. Unfortunately, regulatory restrictions, variability in cannabinoid available formulations (pure CBD, broad-spectrum and full-spectrum extracts, synthetic cannabinoids), and ethical concerns in veterinary clinical trials could impact the feasibility of such research and the obtained results.

Due to the paucity of studies in companion animals, no guidelines or recommendations on how to utilize the endocannabinoid system or cannabinoids to treat cancer in dogs or cats have been provided so far. However, there are anecdotical reports from both human and veterinary oncologists suggesting that the most effective method for the therapeutic use of cannabinoids in oncological patients involves the oral administration of a combination of THC and CBD, generally in a 1:1 ratio. Although such testimonies are mostly based on anecdotal data and to date the oral administration of these substances is not considered the most advantageous, it is believed that this combination could enhance the antitumor effect of the formulation, as the two cannabinoids act through similar yet distinct mechanisms, operating synergistically. In fact, the approach based on using formulations that combine CBD and THC in varying proportions allows for maximizing the antineoplastic action of the treatment while simultaneously reducing the amount of THC needed to inhibit tumor growth. Additionally, CBD helps to mitigate the psychoactive action of THC in both humans and dogs [79].

Results of the veterinary studies cited in this paragraph are summarized in Table 2.

## 6. Conclusions

Cannabinoids show promising antitumor effects in preclinical studies, but clinical evidence remains scarce, particularly in veterinary oncology.

To date, the clinical use of cannabinoids in veterinary medicine is still limited to chronic pain control and palliative care for osteoarticular and neoplastic diseases. Regarding their analgesic efficacy, several studies have been conducted in companion animals, with dogs being the most studied species so far [3,4]. From the randomized, placebo-controlled, double-blind clinical trials conducted to evaluate the therapeutic effects of different cannabis formulations containing mainly CBD in dogs with chronic osteoarthritic (OA) pain and impaired mobility, the treatment significantly reduced pain and increased locomotor activity [80,81,82,83,84,85] or pain from an unknown origin [86]. Moreover, two pilot studies [87,88], a non-blinded observational study [89], and two case reports [90,91] also reported the positive therapeutic effects of CBD administration on OA pain and locomotion. Regarding the feline species, the analgesic efficacy of products containing cannabis derivatives was tested in a placebo-controlled trial conducted in cats with chronic gingivostomatitis [92], where the intervention was positively associated with improved Stomatitis Disease Activity Index (SDAI) score and a marked relief of oral pain. The same was found in a case report on a cat with chronic osteoarthritic pain [93], where pain scores were drastically decreased after treatment. A third study is the one by Hazzah [78] in a feline with advanced cancer, already described in the previous section of this manuscript.

Interestingly, the long-term tolerability of hemp broad-spectrum extracts containing mainly CBD, has been ascertained in dogs and cats [94,95].

Although the use of cannabinoids has been widely documented in palliative medicine, clinical studies on their application as antitumor drugs are still ongoing and far from being representative, especially on veterinary patients. Despite numerous studies on the antitumor activity of cannabinoids, most have been conducted in vitro and in xenograft animal models. While these preclinical studies can be considered numerous enough to realistically believe that the ECS components actively participate in the antitumor response and have a central role in the immune response, their clinical applications in human and especially veterinary medicine can only be considered at their infancy. Indeed, to date, investigations in more complex animal models (such as transgenic animals), where it is possible to reconstruct tumor architecture and the role of the tumor microenvironment and immune response, rendering them representative of the pathological situation of patients, are extremely limited. Moreover, the greatest challenge is the transition from preclinical models to effective clinical use, mainly due to difficulties in designing clinical trials, variability in cannabinoid preparations, and species differences.

If a prolonged variation of the normal endocannabinoid tone, as well as an aberrant expression of its components, has been identified and illustrated in some types of tumors in the canine species (multicentric B and T lymphoma, cutaneous mastocytoma [9,18,19]), the interaction of the ECS with the tumor microenvironment is a field of research that still shows some points that need to be clarified. Immune cells within this habitat express receptors and enzymes belonging to the ECS, so much so that some authors speak of an “immune-endocannabinoid system” [31]. The role of cannabinoids in modulating tumor immunity remains one of the most unresolved areas of investigation; however, their capacity to influence immune cell behavior sustains the potential for therapeutic synergy between cannabinoids and immunochemotherapeutic agents, which constitute a key component of emerging strategies in modern oncology. Certainly, a better understanding of this interaction could lead to novel therapeutic strategies that progress beyond direct tumor cell targeting.

From the above paragraphs, it can be concluded that cannabinoids show antitumor activity (decrease in tumor growth and invasiveness) in numerous cell lines and in various animal models of cancer, and that, although clinical studies conducted in human and animal patients are limited, the results obtained so far have demonstrated that cannabinoids appear to be safe and effective antineoplastic agents. Moreover, most of the preclinical evidence currently available demonstrates that the greatest therapeutic potential of cannabinoids lies in their combination with existing chemotherapeutic drugs. However, many questions remain unanswered, and further clinical studies are necessary to confirm or deny these features and to investigate the potential interaction of cannabinoids with other substances (in particular, with other cytotoxic agents), the most effective routes of administration, and, given the immunosuppressive potential of these molecules, their role as pro-tumorigenic.

Interestingly, compared to conventional antineoplastic drugs, which have a plethora of side effects, cannabinoids (especially the non-psychoactive ones, such as CBD) have a broad safety margin. Despite this, the psychoactive properties of some of their components, primarily THC, combined with the general lack of knowledge about these active principles (mainly related to dosing standardization and formulation challenges), as well as legal prescription requirements, limit their use and make many human and veterinary medical practitioners reluctant to prescribe cannabinoid-based therapies. Therefore, while cannabinoids hold significant therapeutic promise, especially given their favorable safety profile, overcoming regulatory hurdles, expanding clinical research, and increasing practitioner education are essential steps to fully integrate these compounds into mainstream human and veterinary medicine.

## Figures and Tables

**Table 1:** Studies published so far reporting the modifications of the endocannabinoid system in the context of canine neoplastic diseases.

**Tumor Type**	**Results**	**Reference**
B-cell and T-cell multicentric lymphoma	Significantly higher levels of OEA, AEA, and PEA in both lymphomas	[9]
Low-grade canine mast cell tumors High-grade canine mast cell tumors	High expression of CB1R and CB2R, at levels like those in healthy mast cells CB1R and CB2R scarcely present	[18]
Low-grade cutaneous canine mast cell tumors (grades 1 and 2)	High CB2R immunoreactivity, and correlation between high CB2R immunoreactivity, absence or light expression of the p21 protein, and strong immunoreactivity of matrix metalloproteinase-1 (MMP-1)	[19]

**Table 2:** Preclinical and clinical studies on the antineoplastic effects of cannabinoids published so far in dogs and cats.

**Preclinical Studies **		
**Cell Line and Treatment**	**Efficacy**	**Reference**
Canine glioma cell lines treated with CBD extracts and purified CBD to evaluate potential cytotoxicity	Purified CBD reduced proliferation and induced cell death via caspase activation	[69]
Malignant canine B-cell lymphoma (1771 and CLB-L1) and T-cell lymphoma (CL-1) cell lines treated with CBD, THC, and WIN 55-212-22 to evaluate the viability of canine non-Hodgkin’s lymphoma cell lines	CBD was the most potent pCB in reducing lymphoma cell viability in 1771 and CL-1 cell lines (based on the IC50 values)	[70]
Canine prostate carcinoma cell lines treated with cannabis extract oils rich in CBD and THC to evaluate the in vitro antitumor effect	CBD- or THC-rich extracts inhibited the proliferation of two canine prostatic carcinoma cell lines (PC1 and PC2)	[71]
**Clinical Studies**		
**Case Report**	**Efficacy**	**Reference**
Cat with a sarcoma located at the eye bulb that recurred after surgical excision, treated exclusively with oral cannabis oil to attempt to reduce the patient’s pain	The mass decreased in size following two weeks of treatment	[77]
Cat with a large mass encompassing most of the cranial thorax and mid-thorax treated with a complex-spectrum cannabis product aimed to reduce cancer-related symptoms	Resolution of cancer-related clinical signs (dyspnea, coughing, and lethargy) for 6 months	[78]

## Data Availability

No new data were created or analyzed in this study. Data sharing is not applicable to this article.

## Abbreviations

The following abbreviations are used in this manuscript:

ECS	Endocannabinoid system
eCB	Endocannabinoids
pCB	Phytocannabinoids
CB1R	Cannabinoid receptor type 1
CB2R	Cannabinoid receptor type 2
TRPV	Transient receptor potential vanilloid
GPR55	G protein-coupled receptor 55
MMP	Matrix metalloproteinase
AEA	Anandamide
2AG	2-arachidonoyoglycerol
OEA	Oleoylethanolamide
PEA	Palmitoylethanolamide
THC	Δ^9^-tetrahydrocannabinol
CBD	Cannabidiol
PKB	Protein kinase B
ROS	Reactive oxygen species
FAAH	Fatty acid amide hydrolase
MAGL	Monoacylglycerol lipase
VEGF	Vascular Endothelial Growth Factor
FGF-2	Fibroblast Growth Factor 2
ICAM-1	Intercellular adhesion molecule 1
TIMP-1	Tissue inhibitor of matrix metalloproteinases-1
Th	T helper
IFN-γ	Interferon gamma
IL	Interleukin
OS	Osteosarcoma
ASCO	American Society of Clinical Oncology
